# Dufulin Impacts Plant Defense Against Tomato Yellow Leaf Curl Virus Infecting Tomato

**DOI:** 10.3390/v17010053

**Published:** 2024-12-31

**Authors:** Liping Huang, Yingying Tang, Shuaixin Wang, Jianbin Chen, Jiao Du, Shuo Yan, Deyong Zhang, Xiaobin Shi, Yong Liu, Fan Li

**Affiliations:** 1 State Key Laboratory for Conservation and Utilization of Bio-Resources in Yunnan, Yunnan Agricultural University, Kunming 650201, China; hlp1936580786@hnu.edu.cn; 2 Institute of Plant Protection, Hunan Academy of Agricultural Sciences, Changsha 410125, China; wangshuaixin1@126.com (S.W.); chenjianbin89@126.com (J.C.); dujiao1234xy@163.com (J.D.); yanshuo189@hotmail.com (S.Y.); dyzhang78@163.com (D.Z.); 3 MARA Key Laboratory of Sustainable Crop Production in the Middle Reaches of the Yangtze River (Co-Construction by Ministry and Province), Yangtze University, Jingzhou 434025, China; 18976586507@163.com; 4 Yuelushan Laboratory, Changsha 410125, China

**Keywords:** TYLCV, dufulin, *NPR1* gene, *PI II* gene, chlorophyll, nitrogen

## Abstract

*Tomato yellow leaf curl virus* (TYLCV) poses a significant threat to tomato production, leading to severe yield losses. The current control strategies primarily rely on the use of pesticides, which are often nonselective and costly. Therefore, there is an urgent need to identify more environmentally friendly alternatives. Dufulin, a novel compound that has been effective in controlling viral diseases in tobacco and rice, has not yet been tested against TYLCV. This study assessed the efficacy of dufulin in controlling TYLCV over a three-year span from 2021 to 2023 through field trials, by monitoring disease symptoms and viral titers. Additionally, this study assessed the expression levels of genes associated with systemic acquired resistance (SAR), specifically *proteinase inhibitor II* (*PI II*) and *non-expressor of pathogenesis-related genes 1* (*NPR1*), using real-time qRT-PCR. The chlorophyll and nitrogen content in the leaves were also measured. Plants treated with dufulin showed reduced symptomatology and lower viral titers compared to the controls. Analysis of gene expression revealed that *NPR1* was upregulated in the dufulin-treated plants, whereas *PI II* expression was consistently downregulated in the TYLCV-infected plants. Interestingly, *PI II* expression increased in the healthy plants following a seven-day post-treatment with dufulin. Moreover, the treated plants exhibited a higher chlorophyll content than the controls, though no significant differences in the nitrogen levels were observed between the dufulin-treated and water-treated plants. Overall, the application of dufulin significantly bolstered the plant’s defense response, effectively reducing TYLCV symptoms and enhancing resistance.

## 1. Introduction

*Tomato yellow leaf curl virus* (TYLCV), a member of the genus *Begomovirus* within the family Geminiviridae, is a plant pathogen of significant economic concern. It is transmitted by the sweet potato whitefly *Bemisia tabaci* species complex in a persistent, circulative manner in the field [1,2,3]. TYLCV-infected tomato plants exhibit symptoms such as leaf yellowing, curling, and stunting, which collectively lead to substantial reductions in tomato yields worldwide [4,5]. In addition to tomatoes, TYLCV affects other major crops, including peppers, common beans, and cucurbits [6,7,8]. Managing TYLCV effectively is widely recognized as a notorious challenge, whether in greenhouses or open fields, with traditional approaches often proving inefficient [9]. Various control strategies, including vector removal, weed elimination, and timing adjustments for planting, have been employed with varying degrees of success [2,9]. TYLCV-resistant commercial tomato cultivars have also been developed to combat TYLCV infections so far [10,11]. However, the virus’s high genetic variability frequently overcomes these defenses, compromising their long-term effectiveness [12,13]. While treatments relying on chemicals and pesticides have been explored, their associated environmental and health risks often limit their desirability [14]. This highlights the importance of seeking safer, more sustainable alternatives, such as the novel antiviral agent dufulin.

Dufulin is an amino phosphonate compound with a novel molecular structure ((2-fluorophenyl)-(((4-methylbenzothiazol-2-yl)-amino) methyl)) phosphonic acid diethyl ester [15,16]. It is the first antiviral agent in China to comply with environmental safety standards, demonstrating remarkable efficacy in controlling plant viruses [17,18]. Initially identified in 2007 for its potent anti-TMV activity by the Ministry of Agriculture of China (LS 20071280 and 20071282), dufulin has been shown to induce systemic acquired resistance (SAR) in tobacco plants [15,16,19]. Other than on tobacco, SAR-inducing activity was also observed when dufulin was applied to plants infected by southern rice black-streaked dwarf virus (SRBSDV) [20]. Another study also indicated that dufulin actively inhibits viral virulence in SRBSDV by binding to specific viral proteins, such as arginine 175 in the P9-1 octameric protein, and targeting viroplasmic protein P6 [21,22]. The dual modes of action have established dufulin as an effective tool for controlling viral diseases in tobacco and rice, and its large-scale industrial production has facilitated its broad application in field conditions [23,24].

Given dufulin’s success against other plant viruses, we hypothesized that dufulin could also be effective against TYLCV, a virus that poses a persistent threat to global tomato production. To validate this hypothesis, we conducted field experiments spanning three planting cycles (2021–2023) to assess dufulin’s efficacy in controlling TYLCV. Furthermore, given evidence of dufulin-induced SAR activation in tobacco [19,25], we investigated its potential to activate similar mechanisms in tomatoes by analyzing SAR-related gene expression and leaf nutritional properties. We focused on two SAR-related genes: *proteinase inhibitor II* (*PI II*) and *non-expressor of pathogenesis-related genes 1* (*NPR1*). PI II is regulated by jasmonic acid (JA) signaling, which is triggered in response to insect attacks and enhances resistance in solanaceous plants, including tomatoes [26,27]. In contrast, NPR1 is a critical regulator of SAR mediated by salicylic acid (SA) signaling, which bolsters plant immunity against broad-spectrum pathogens [28,29].

This study aimed to determine dufulin’s impact on both healthy and TYLCV-infected tomato plants, and to uncover the mechanisms underlying its effects. By evaluating dufulin’s capacity to suppress TYLCV and elucidating the involved pathways, our findings will contribute to the development of sustainable and environmentally friendly strategies for managing TYLCV in tomato cultivation.

## 2. Materials and Methods

### 2.1. Field Control Effect of Dufulin on TYLCV

The field experiment was conducted in the Chunhua vegetable-growing area of Changsha City, Hunan Province, China, where TYLCV disease is consistently observed annually. Non-resistant tomato plants to TYLCV (*Solanum lycopersicum*, cultivar Zuanhongmeina, Hunan Academy of Agricultural Sciences) were transplanted at the six-true-leaf stage each April, spanning from 2021 to 2023. The plants were healthy at the time of transplantation. The field environment maintained an average temperature range of 14–22 °C (source: World Weather Online). Within the first three days post-transplantation, any dead plants were replaced to ensure uniformity among the treatment groups.

Two treatments were compared: one with dufulin application and the other with water-only as a control. The dufulin treatment utilized a 20% (effective components) suspension prepared according to the manufacturer’s instructions (Guangxi Garden Biochemical Joint Stock Company, Nanning City, Guangxi, China). Each treatment was replicated three times, yielding six plots per planting cycle. The plots were arranged in a randomized design, with each plot containing 60 tomato plants. Specifically, each plot was composed of 15 rows, with 4 plants per row, and protective buffer rows were established between the plots. No other antiviral agents were applied to the field before or during the experiment to avoid interference with the treatments.

**Figure 1:**
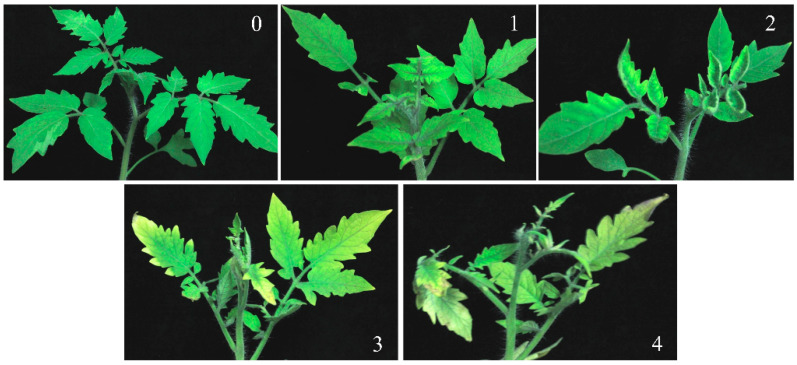
The severity rating scale for *tomato yellow leaf curl virus* (TYLCV) symptoms in tomato plants. The scale is defined as follows: 0 = asymptomatic; 1 = slight symptoms, characterized by mild yellowing of leaflet margins on apical leaves; 2 = moderate symptoms, with some yellowing and minor curling of leaflet ends; 3 = severe symptoms, marked by marginal or interveinal yellowing and curling of leaves, along with a noticeable reduction in plant size; 4 = very severe symptoms, including extreme yellowing, curling, and stunting, which ultimately halt its growth.

Treatments were applied 30 days after transplantation using a 16-L AGROLEX SPRAYER JACTO HD400 knapsack sprayer (Linon Private Limited Company, Singapore). The sprayer operated at a pressure of 1–2 kgf/cm^2^ with a flow rate of 255–950 g/min. Each plot received a uniform application of 5 L of dufulin solution, and treatments were repeated every 7 days for a total of three applications. All applications were conducted on sunny days, and plants were monitored for signs of phytotoxicity two days after each spray. Disease incidence was assessed 14 days after the final application. A total of 360 plants were evaluated across all plots. Each plant was assigned a disease severity level according to established criteria from previous studies [30,31]. Plants with a severity level above zero were categorized as morbid. The distribution of disease levels is visually illustrated in Figure 1. The disease index and control effect were calculated by the following formulas:

Disease index = Σ (Number of diseased plants at every level × Level value)/(Total survey plants × the highest value)(1)

Control effect = [(Disease index of the control group − Disease index of the treatment group)/Disease index of the control group] × 100%(2)

### 2.2. TYLCV Infection Rates in Tomato Plants of Disease Symptoms on the Field

The objective of this study was to ascertain whether disease symptoms in young tomato plants were related to TYLCV. We randomly collected 20 individuals of tomato plants with different degrees of yellowing and curling (characteristic symptoms of TYLCV infection) on the field in 2021 and returned leaves to the lab for TYLCV detection via PCR. Tomato leaves’ total DNA was extracted using 2 × CTAB extraction buffer (Solarbio Science & Technology Co., Ltd., Beijing, China) according to the instructions. The DNA was used as a template for PCR amplification with the primers TYLCV-F/R (Table 1) by Green Taq Mix (Vazyme, Nanjing, China). The PCR products were detected by agarose gel electrophoresis and sent to Sangon Biotech (Shanghai, China) for sequencing.

### 2.3. TYLCV Titer from the Field Experiment with TAS-ELISA

After investigating the plant’s morbidity, tomato leaves were sampled by a 5-point diagonal sampling method (samples were taken from the four corners of the field and the intersection stagnation point of two diagonals, 2 plants at each point), which resulted in 10 samples per treatment. The leaves were tested for TYLCV presence with a triple antibody sandwich enzyme-linked immunosorbent assay (TAS-ELISA). The plant TYLCV TAS-ELISA kit (Adgen Phytodiagnotics, Neogen Coporation, Michigan, United States), utilizing anti-TYLCV coat protein (CP) antibodies, was used to conduct the assay [32,33]. Three leaves’ sample of 0.1 g per plant (one each from the top, middle, and bottom of the plant) was ground in 1 mL of extraction buffer, then centrifuged at 3000 rpm for 20 min at 4 °C; the clarified plant sap was incubated with the antibody at 37 °C for 1 h, following the manufacturer’s instructions. Absorbance was read with a spectrophotometer at the wavelength of 405 nm. Since the past study had established that OD data are linked to the accumulation of virus in a plant infected with the virus, the sample was considered positive for TYLCV when the mean optical density (OD) values were over three times those of healthy tomato plants grown in a laboratory greenhouse.

### 2.4. Effects of Dufulin on SAR-Related Gene Expression and Tomato Leaves’ Nutrition

Preparation. Tomato (*Solanum lycopersicum*, Zuanhongmeina) plants were grown in pots (d = 12.5 cm, h = 15 cm) and kept in whitefly proof screen cages (60 × 40 × 60 cm) in a greenhouse under a photoperiod of L:D 16 h:8 h, temperature of 25 ± 1 °C, and relative humidity of 60 ± 10%. To create TYLCV-infected tomato plants, a 0.5 mL TYLCV infectious DNA *Agrobacterium* clone (pCambia2300-TYLCV-BJ-1.4DNA) was injected into the four-true-leaf stage tomato plant. Both visual (leaf yellowing and curling) and molecular (PCR) inspections were conducted to confirm the presence of viral infection [34]. The primers used to detect TYLCV are in Table 1. For the experiment, a set of healthy and TYLCV-infected tomato plants, each 30 days old, were treated with either dufulin solution (treatment) or water (control). Each treatment and control group included nine plants. Approximately 15 mL of dufulin solution was applied to each plant in the treatment group, while the control group received an equal volume of water. Following the treatments, the plants were evaluated for SAR-related gene expression and leaf nutrition content.

Measurement of SAR-related genes *PI II* and *NPR1* expression in tomato plants. From both healthy and TYLCV-infected plants, 0.1 g of the third fully expanded leaf from the top of the plant was collected daily into RNase-free sampling tubes. Fully expanded leaves from the same lateral branches were sampled over a seven-day period, starting on the first day after spraying. All collected leaves were immediately stored at −80 °C for subsequent analysis. Total RNA was extracted using Transzol Up (TransGen Biotech, Beijing, China), and RNA concentration and purity were assessed with a NanoDrop 2000 spectrophotometer (Thermo Fisher Scientific, Beijing, China).

Real-time fluorescent quantitative reverse transcriptase polymerase chain reaction (qRT-PCR) was used to measure the expression levels of jasmonic acid (JA)- and salicylic acid (SA)-related genes (*PI II* and *NPR1*) in tomato plants following treatment. The qPCR primers for these SAR-related genes are listed in Table 1. An amount of 400 ng of RNA was used to synthesize the first-strand cDNA with the HiScript^®^ II 1st Strand cDNA Synthesis Kit (+gDNA wiper) (Vazyme, Nanjing, China) according to the manufacturer’s instructions. qRT-PCR was performed on a qTOWER3G system (Analytik Jena, Jena, Germany) using ChamQ^®^ Universal SYBR qPCR Master Mix (Vazyme, Nanjing, China). The 20 μL reaction mixture included 8.2 μL of RNase-free water, 1 μL of cDNA, 10 μL of SYBR qPCR master mix, and 0.4 μL each of 10 μM forward and reverse primers. Gene expression levels were normalized using the 2^−ΔΔCT^ method [35], with *Actin* (*ACT*) and *Ubiquitin* (*UBI*) as reference genes [36]. The geometric mean of the Ct values for the *ACT* and *UBI* genes was used to calculate the 2^−ΔΔCT^ values for the target genes.

Measurement of chlorophyll and nitrogen content in tomato leaves. Chlorophyll and nitrogen levels were assessed as nutritional markers to evaluate the overall health of the plants. A handheld chlorophyll and nitrogen meter (Okechi Instrument Company, Zhengzhou City, Henan, China) was used for the measurements, which were consistently conducted on the third fully expanded leaf from the top of each plant. The same leaves were monitored over a seven-day period.

### 2.5. Data Analysis

Data analysis was performed using SPSS version 20.0 (SPSS Inc., Chicago, IL, USA). An independent samples *t*-test was conducted to evaluate the field control efficacy of dufulin. One-way ANOVA was used to compare TYLCV titers in tomato plants. A general linear model (GLM) with repeated measures was applied to analyze the effects of dufulin on plant defense gene expression and nutritional content. GLM results are reported in the format F (df between treatments, df within treatments). Statistical significance was defined as a *p*-value less than 0.05.

## 3. Results

### 3.1. TYLCV Infection Rates in Tomato Plants

**Figure 2:**
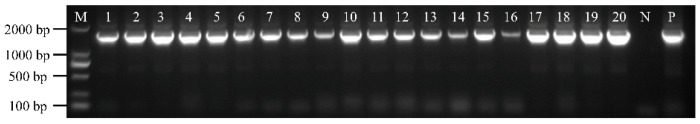
TYLCV infection rates in tomato plants displaying disease symptoms in the field. M: DNA marker; 1–20: 20 individual tomato plants with leaves exhibiting varying degrees of yellowing and curling in the field; N: negative control (healthy tomato leaf); P: positive control (TYLCV-infected tomato leaf).

Specific primers (TYLCV-F/R) were used to detect TYLCV infection in 20 tomato plants that exhibited varying degrees of leaf yellowing and curling in the field. PCR amplification yielded a ~1600 bp fragment, as confirmed by agarose gel electrophoresis, which matched the expected target size (Figure 2). Sequencing results were subjected to BLAST analysis on NCBI (https://www.ncbi.nlm.nih.gov/, accessed on 24 June 2021), revealing 99.0% homology with the reference sequence KY499720.1 for TYLCV. While the possibility of co-infection with other viruses in the field cannot be definitively ruled out, the symptoms of yellowing and curling in the tomato plants can be unequivocally attributable to TYLCV.

### 3.2. Field Control Effect of Dufulin and TYLCV Titer in Tomato Plants

**Figure 3:**
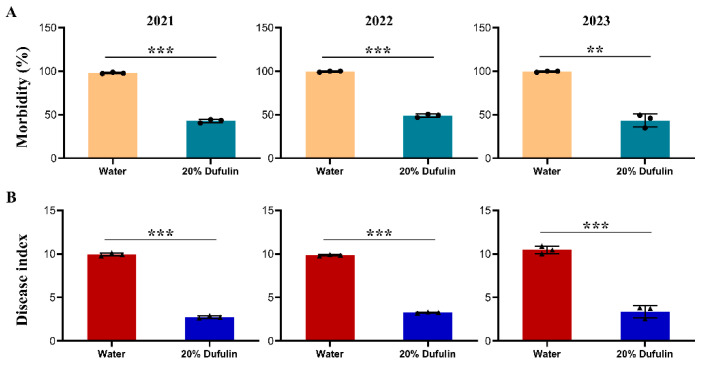
The morbidity and disease index of tomato plants in field experiments from 2021 to 2023. (**A**) The morbidity; (**B**) the disease index. Data were pooled with three replications; Data are given as mean ± SD; the asterisks above bars mean significant difference: ** *p* < 0.01, *** *p* < 0.001.

Field experiments revealed that tomato plants treated with 20% dufulin exhibited an average morbidity of 42.85%, 48.92%, and 43.36% in the 2021, 2022, and 2023 planting cycles, respectively. These rates were notably lower than those of the control group (water spray only), which consistently showed morbidity rates above 97% across all planting cycles (2021, t (4) = 42.862, *p* < 0.001; 2022, t (4) = 42.137, *p* < 0.001; 2023, t (4) = 12.751, *p* = 0.006) (Figure 3A). Morbidity represents the proportion of plants exhibiting TYLCV symptoms within each group. Furthermore, the dufulin-treated plants had a significantly lower disease index compared to the control group (2021, t (4) = 56.671, *p* < 0.001; 2022, t (4) = 115.749, *p* < 0.001; 2023, t (4) = 14.695, *p* < 0.001). The disease index for the dufulin-treated plants was 2.75, 3.27, and 3.37, lower than that of the control group in 2021, 2022, and 2023, respectively (Figure 3B). Overall, the average field control effect of the 20% dufulin solution, calculated using Equation (2), was 68.29%, making it more effective than the control over the three planting cycles.

When the tomato leaves were analyzed for TYLCV presence, the mean optical density (OD) values measured at 405 nm for both the 20% dufulin-treated and water-only groups were over three times higher than those of the healthy plants, confirming the presence of TYLCV in the leaves. However, the TYLCV titers were significantly lower in the dufulin-treated plants compared to the water-treated controls across all planting cycles from 2021 to 2023 (2021, F (2, 27) = 1186.36, *p* < 0.001; 2022, F (2, 27) = 1836.211, *p* < 0.001; 2023, F (2, 27) = 1262.753, *p* < 0.001) (Supplemental Figure S1). These results indicate that dufulin treatment effectively suppresses TYLCV titers in tomato leaves compared to water treatment alone.

### 3.3. Change in PI II and NPR1 Gene Expression in Tomato Plants

**Figure 4:**
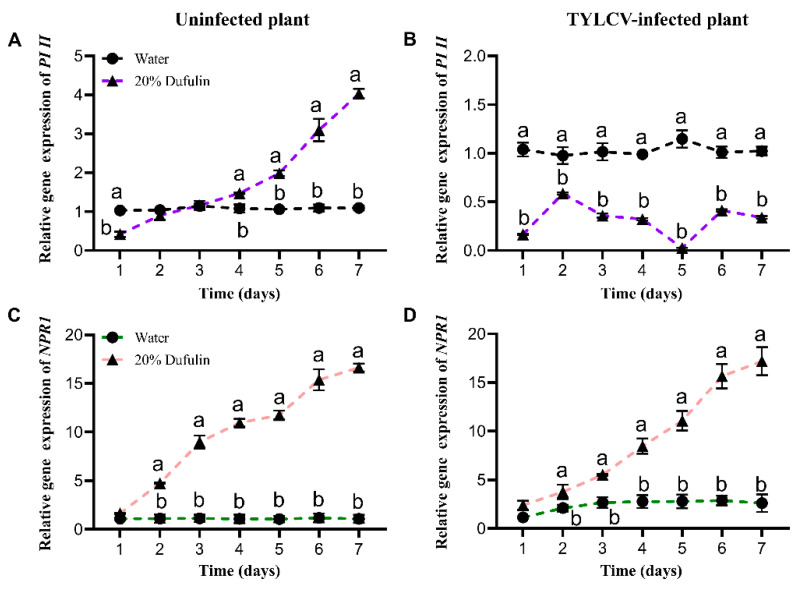
The relative expression level of *PI II* and *NPR1* in tomato plants after spraying with 20% dufulin and water for days 1, 2, 3, 4, 5, 6, and 7. (**A**,**C**): In uninfected tomato plants. (**B**,**D**): In TYLCV-infected tomato plants. Data were pooled with three replications; Data are given as mean ± SD. Different letters indicate significant differences (*p* < 0.05).

The expression patterns of the *PI II* and *NPR1* genes are shown in Figure 4. *PI II* expression displayed distinct trends between the uninfected and TYLCV-infected plants. In the healthy plants, *PI II* expression significantly differed between those treated with 20% dufulin and the water-treated controls, except on days 2 and 3 (F (1, 20) = 156.275, *p* < 0.05). Additionally, the dufulin-treated healthy plants showed a consistent increase in *PI II* expression over time (Figure 4A). In the TYLCV-infected plants, however, *PI II* expression was significantly lower in the dufulin-treated samples compared to the water-treated controls (F (1, 20) = 333.499, *p* < 0.05). Furthermore, in infected plants treated with dufulin, *PI II* expression progressively declined after day 2, whereas the expression levels in the water-treated controls remained relatively stable (Figure 4B).

Our analysis of *NPR1* expression revealed a steady increase over seven days in both the TYLCV-infected and uninfected plants compared to the control levels. In the healthy plants, *NPR1* expression was significantly elevated (Figure 4C; F (1, 20) = 9713.138; *p* < 0.05), as it was in the TYLCV-infected plants (Figure 4D; F (1, 20) = 472.545; *p* < 0.05), reaching 15- to 17-fold higher levels than the control by day 7 in both groups. Additionally, in the water-treated plants, *NPR1* expression was higher in the TYLCV-infected plants than in the healthy ones, consistent with typical salicylic acid (SA) signaling activation during pathogen attack, although this difference was not statistically significant.

### 3.4. Change in Chlorophyll and Nitrogen Content in Tomato Plants

**Figure 5:**
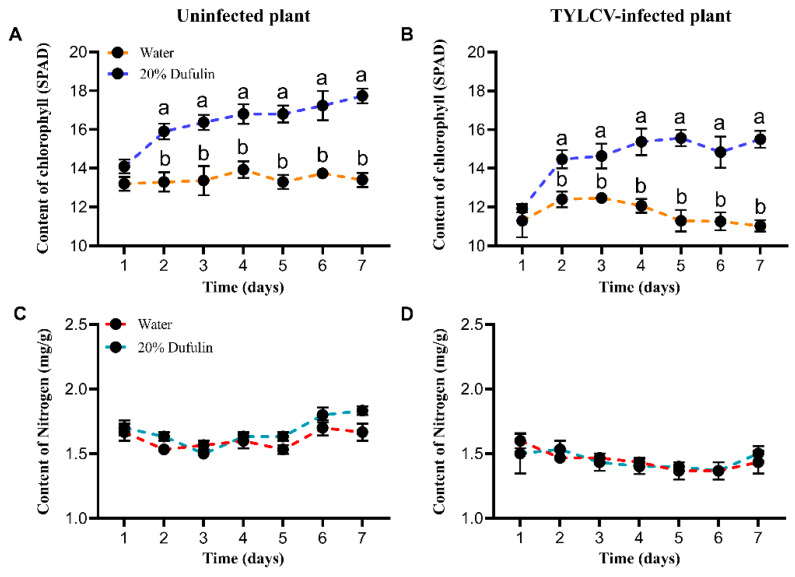
The content of chlorophyll (SPAD) and nitrogen (mg/g) in tomato plants after spraying 20% dufulin and water for days 1, 2, 3, 4, 5, 6, and 7. (**A**,**C**): In uninfected tomato plants. (**B**,**D**): In TYLCV-infected tomato plants. Data were pooled with three replications; Data are given as mean ± SD. Different letters indicate significant differences (*p* < 0.05).

In terms of chlorophyll content, the dufulin-treated plants, whether TYLCV-infected or uninfected, exhibited significantly higher chlorophyll levels than the water-treated plants, except on day 1 (uninfected plant: F (1, 20) = 137.385, *p* < 0.05; TYLCV-infected plant: F (1, 20) = 88.042, *p* < 0.05). Over the seven-day period, the chlorophyll content gradually increased in the dufulin-treated plants compared to the controls (Figure 5A,B). Regarding the nitrogen content, no significant differences were observed between the dufulin- and water-treated plants, either in the uninfected (F (1, 20) = 4.170) or in the TYLCV-infected plants (F (1, 20) = 5.256) (Figure 5C,D). Furthermore, the chlorophyll and nitrogen content were higher in the uninfected plants compared to the TYLCV-infected plants, regardless of whether they were treated with dufulin or water (Figure 5).

## 4. Discussion

For several years, dufulin has been widely used to prevent and control viral diseases in crops such as tobacco and rice. Extensive research has been conducted to examine its control efficacy, mechanisms of action, environmental biotoxicity, and safety evaluation, particularly regarding its role in enhancing plants’ innate resistance to viral infections [25,37]. Currently, TYLCV poses a significant threat to tomato production, causing severe damage and yield losses. This study represents the first attempt to apply dufulin against TYLCV in tomato plants. Our three-year field study demonstrated that dufulin application in plants 30 days after transplanting to the field can effectively mitigate TYLCV symptoms, as evidenced by a reduction in morbidity and viral titers compared to the water-treated controls (Figure 3 and Supplementary Figure S1). Approximately 50% of the dufulin-treated plants remained asymptomatic, and the ELISA titer results confirmed that dufulin reduced the viral loads in the infected plants. These findings suggested that dufulin may induce the plant to keep the TYLCV population from reaching a point that causes severe symptoms and plant growth impairment.

Gene expression analyses provided further insights into the mechanism of action of dufulin. We observed a significant upregulation of the *NPR1* gene, a key regulator of systemic acquired resistance (SAR). Upon activation by the accumulation of salicylic acid (SA), NPR1 functions as a co-transcription factor that translocates to the nucleus, thereby inducing the expression of pathogenesis-related genes [38,39]. This mechanism establishes long-term SAR in plants. Our study aligns with previous findings showing increased *NPR1* expression under pathogen attack [29,40,41]. Interestingly, the dufulin-treated plants exhibited similar *NPR1* expression levels in both the healthy and TYLCV-infected plants by the end of the observation period (Figure 4C,D), suggesting that dufulin strongly activates the SA pathway, irrespective of the infection status. Although the exact mechanism by which dufulin induces *NPR1* overexpression remains unclear, the findings from Chen et al. [25] on tobacco suggest that dufulin activates harpin-binding protein 1 (HrBP1), leading to SA accumulation. Given the presence of HrBP1 in tomatoes [42], it is plausible that a similar pathway is involved here. However, further studies are needed to confirm this hypothesis, particularly regarding the interplay between dufulin and SA signaling in tomatoes.

Our study also examined the expression of the *PI II* gene, a marker for the jasmonic acid (JA) pathway, which is typically activated in response to herbivory [43,44]. Interestingly, dufulin treatment caused differential *PI II* expression between the healthy and TYLCV-infected plants. In the water-treated controls, *PI II* expression remained comparable between the healthy and infected plants, suggesting that TYLCV does not naturally induce this gene (Figure 4A,B). In the dufulin-treated plants, however, *PI II* expression was suppressed in the infected plants but slightly elevated in the healthy plants. This suppression may result from antagonistic crosstalk between the SA and JA pathways, as SA accumulation—induced by dufulin—can inhibit JA signaling [45,46,47,48]. The virus may further modulate this interaction, leading to the observed suppression of *PI II* in the infected plants. Additional research is needed to unravel the regulatory mechanisms governing this response. One limitation of our gene expression analysis is that we excluded insect vectors, introducing the virus directly into the plants. Studies by Zhang et al. [49] have shown that whitefly feeding can influence JA pathway activation, potentially benefiting whitefly nymphs by suppressing plant defenses. However, TYLCV infection may counteract this benefit by reducing whitefly fecundity and abundance [50]. While our field experiments included natural tritrophic interactions (plant–virus–vector), future studies should incorporate these interactions into gene expression analyses to better mimic real-world conditions.

Regarding the nitrogen and chlorophyll content, dufulin treatment had no significant effect on the leaf nitrogen levels (Figure 5C,D). However, the chlorophyll content—a nitrogen-containing molecule—was consistently higher in the dufulin-treated plants (Figure 5A,B). This finding aligns with previous studies linking higher chlorophyll levels to increased TYLCV resistance [51]. Additionally, chlorophyll content serves as an important indicator of plant nutritional status and can guide fertilization strategies to improve crop quality and yield [52,53]. Dufulin may enhance chlorophyll production by reallocating nitrogen resources to support photosynthesis and defense mechanisms, but further studies are needed to elucidate this process.

While our study sheds light on dufulin’s role in inducing SAR and its impact on chlorophyll and nitrogen content, several questions remain unanswered. For instance, the specific receptors and signaling pathways involved in dufulin’s action, as well as its potential direct antiviral properties, require further investigation. Evidence from studies on SRBSDV suggests that dufulin may act as an active virucide, raising the possibility that it could have similar effects against TYLCV.

## 5. Conclusions

In this study, we evaluated the efficacy of dufulin against TYLCV in tomato plants over three planting cycles. While its overall field efficacy was moderate compared to active pesticides, dufulin effectively reduced TYLCV symptoms and viral loads, preventing severe infection and disease progression. Its ability to induce SAR, as evidenced by *NPR1* upregulation, further highlights its potential as a plant defense activator. Given these findings, dufulin could be integrated into strategies for tomato disease management, particularly when utilized in conjunction with minimal pesticide applications or biological control agents that target whitefly vectors (e.g., *Bemisia tabaci*). These combined approaches have the potential to promote its adoption as a sustainable and environmentally friendly alternative for controlling TYLCV in tomato crops.

## Figures and Tables

**Table 1:** Primers used in this study.

Names	GenBank Accession No.	Primer Sequence (5′-3′) *
TYLCV	KY499720.1	F: GTTCACGGATTTCGTTGTATG
		R: AGAGGGACTGGCAAAGCAACA
*ACT*	BT013707	F: AGGCAGGATTTGCTGGTGATGATGCT
		R: ATACGCATCCTTCTGTCCCATTCCGA
*UBI*	X58253	F: TCGTAAGGAGTGCCCTAATGCTGA
		R: CAATCGCCTCCAGCCTTGTTGTAA
*PI II*	K03291.1	F: CCTATTCAAGATGTCCCCGTTC
		R: GGGCAATCCAGAAGATGG
*NPR1*	AY640378.1	F: ATATAGAATTCCTGCTCCAAAGGATCGGTTA
		R: ATATACTCGAGCAGACAAGTCATCAGCATCCA

* F indicates forward primer; R indicates reverse primer.

## Data Availability

Data are contained within the article.

## Supplementary Materials

The following supporting information can be downloaded at: https://www.mdpi.com/article/10.3390/v17010053/s1, Figure S1: The TYLCV titer in tomato plants from 2021 to 2023.
