# Supplementary material for: Dufulin Impacts Plant Defense Against Tomato Yellow Leaf Curl Virus Infecting Tomato
Source: Viruses. 2024 Dec 31;17(1):53. doi: 10.3390/v17010053 (PMC11768724; doi:10.3390/v17010053)
Supplement: Supplementary file 1 [file viruses-17-00053-s001.zip › viruses-3368886-supplementary.pdf]

Supplementary materials:

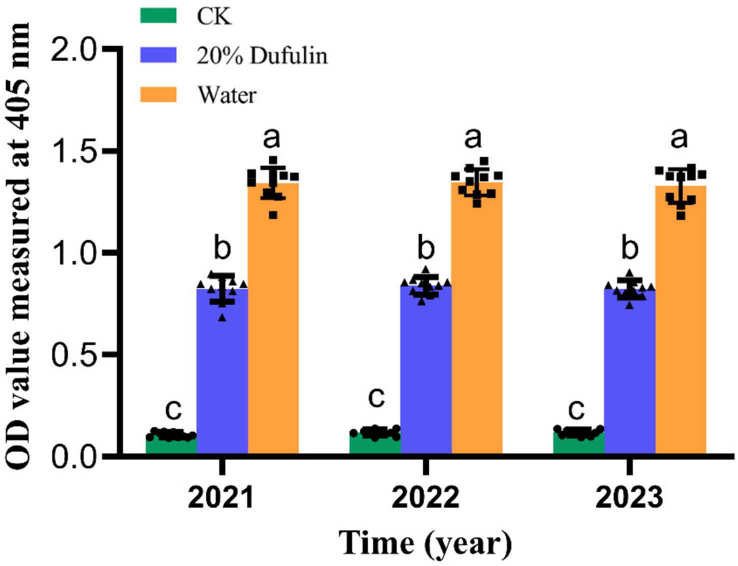

Figure S1 The TYLCV titer in tomato plants from 2021 to 2023. CK: leaves from healthy tomato plants; 20% Dufulin: leaves from 20% dufulin treatments; Water: leaves from water treatments. Data were pooled with 10 replications; Data are given as mean ± SD. Different letters indicate significant differences ($p < 0.001$).
